# Fetal Programming Influence on Microbiome Diversity and Ruminal and Cecal Epithelium in Beef Cattle

**DOI:** 10.3390/ani14060870

**Published:** 2024-03-12

**Authors:** Evandro Fernando Ferreira Dias, Felipe Eguti de Carvalho, Guilherme Henrique Gebim Polizel, Fernando Augusto Correia Queiroz Cançado, Édison Furlan, Arícia Christofaro Fernandes, Fernando José Schalch Júnior, Gianluca Elmi Chagas Santos, José Bento Sterman Ferraz, Miguel Henrique de Almeida Santana

**Affiliations:** 1 Department of Animal Science, Faculty of Animal Science and Food Engineering—USP, Av. Duque de Caxias Norte, 225, Pirassununga 13635-900, SP, Brazil; evandrodias4@usp.br (E.F.F.D.); guilherme.polizel@usp.br (G.H.G.P.); facqc@usp.br (F.A.C.Q.C.); edisonfurlan@usp.br (É.F.); fernando@minerthal.com.br (F.J.S.J.); gianluca1555@usp.br (G.E.C.S.); 2 Department of Veterinary Medicine, Faculty of Animal Science and Food Engineering—USP, Av. Duque de Caxias Norte, 225, Pirassununga 13635-900, SP, Brazil; felipe.eguti@usp.br (F.E.d.C.); jbferraz@usp.br (J.B.S.F.)

**Keywords:** rRNA 16s, metabolism, metagenomics, nutrigenomics, prenatal nutrition

## Abstract

**Simple Summary:**

Fetal programming through different maternal nutritional strategies can impact the development of the rumen and cecum in the offspring of Nellore cattle. We evaluated bulls submitted to different prenatal nutrition strategies, namely, non-programming, partial programming, and complete programming. Rumen epithelium was meticulously evaluated for rumenitis and structural irregularities post-slaughter, while cecal lesions were examined after evisceration. Additionally, DNA extraction and the sequencing of distinct amplicon sequence variants in the rumen ecosystem were undertaken. Our metagenomic analysis provided insights into microbial communities influenced by maternal nutrition and dietary factors. This study advances our understanding of fetal programming by emphasizing the intricate interplay between maternal nutrition, gastrointestinal development, and microbial communities. These discoveries contribute significantly to the field of livestock.

**Abstract:**

We explored the influence of maternal nutritional strategies on the development of the rumen and cecum in offspring. Additionally, we investigated the potential repercussions of prenatal nutrition on the rumen and fecal microbiota composition, utilizing metagenomic 16S techniques, to understand the effects of fetal programming (FP) in Nellore cattle. A total of 63 bulls submitted to different prenatal nutrition strategies, namely, non-programming (NP), partial programming (PP), and complete programming (CP), were evaluated. The rumen epithelium was methodically evaluated based on the presence of rumenitis and structural irregularities. The assessment of cecum lesions was conducted post-evisceration, whereby all thoroughly cleaned ceca were methodically evaluated. Samples from 15 animals of rumen fluid at slaughter and feces during the finishing phase were collected, respectively. All DNA extraction were carried out using the Macherey Nagel NucleoSpin Tissue^®^, and 16S sequencing was conducted using the V4 primers on the MiSeq platform. Within the ruminal ecosystem, an estimated range of 90 to 130 distinct amplicon sequence variants was discerned, as distributed across 45,000 to 70,000 sequencing reads. Our metagenomic exploration unveils microbial communities that distinctly mirror gastrointestinal tract microenvironments and dietary influences. In sum, this comprehensive study advances our comprehension of FP, highlighting the interplay of maternal nutrition, gastrointestinal development, and microbial communities, contributing significantly to the fields of animal science.

## 1. Introduction

In the livestock industry, particularly in beef cattle production, optimizing productivity and product quality is of paramount importance. With Brazil’s significant role in global cattle production, the need to meet both domestic and international demands for high-quality meat have never been more critical [1]. As the world’s second-largest beef producer and the largest exporter, responsible for a substantial portion of the national gross domestic product (GDP) and trade balance, Brazil has a strong interest in advancing its beef production capabilities [2].

A pivotal aspect of enhancing beef production lies in fetal programming (FP), a strategy focused on improving the prenatal environment of developing calves through maternal nutrition [3]. This approach, rooted in the manipulation of maternal nutrition during specific gestational periods, has the potential to influence various aspects of calf development, thereby affecting their entire life cycle [4]. Research has shown that the gestational phase is a crucial period for the development of muscle fibers, an attribute directly linked to meat production [5]. Moreover, it is during this period that microbial colonization begins in the gastrointestinal tract, raising questions about the correlation between the uterine environment and the establishment of these early microorganisms [6].

As FP gains recognition for its impact on muscle, bone, and fat production in cattle, it also extends its influence on other vital areas [7]. This includes the microbiome within the rumen and feces of these animals, which is important in digestion, nutrient utilization, and, ultimately, feed efficiency [4]. Alterations in the uterine environment have the potential to affect the composition and diversity of the gastrointestinal microbiota. These alterations, in turn, may impact not only the nutritional efficiency of the cattle but also the overall productivity and environmental sustainability of the system [8,9]

However, despite the significance of FP in cattle, studies evaluating its effects on the ruminal and cecal epithelium and microbial diversity are relatively scarce. This gap in knowledge is a critical area of inquiry for both the improvement of meat production and the sustainability of the cattle industry. Hence, this study aims to explore the influence of maternal nutritional strategies on the development of the rumen and cecum in offspring. Additionally, we investigated the potential effects of prenatal nutrition on the composition of the rumen and fecal microbiota, utilizing metagenomics to better understand the holistic effects of FP on beef cattle.

## 2. Materials and Methods

### 2.1. Experimental Design

All animals (cows and bulls) used in the current study were sourced from the Fernando Costa Campus, FZEA/USP. Initially, Nellore dams were subjected to fixed-time artificial insemination (FTAI) using semen from four sires. Pregnancy diagnoses were confirmed in February 2018 during the breeding season. Following confirmation, the cows were selected based on age, body weight, and body condition score and subsequently divided into three groups (treatments), each comprising 42 animals.

These animals were then placed in pastures of Urochloa brizantha cv. Marandu with access to bunk feeders and water ad libitum. The distinguishing factor among the groups was the specific nutritional plan provided to each group during pregnancy. These plans were the following treatments programs: (NP) non-programming, where cows received only a mineral supplementation equivalent to 0.3 g/kg of body weight (BW); partially programming (PP), which included supplementation with protein energy supplementation at 3 g/kg of BW during the last third of pregnancy; and complete programming (CP), where the cows received 3 g/kg of BW of protein energy supplementation from the moment of pregnancy confirmation (30 days after FTAI) until birth. For further details on the treatments, see Schalch Junior et al. [10].

After calving, the supply of protein energy supplements was discontinued, and all animals were grouped together, irrespective of their earlier nutritional plan. The animals underwent vaccination against tetanus and bovine viral diarrhea virus, a 7-way *Clostridium* sp., and adhered to health protocols. The progeny was weaned at seven months of age. After weaning, the animals were segregated by gender into males and females, regardless of their treatment group. This rearing phase of 63 male progeny (NP = 22; PP = 20, and CP = 21) was extended for 11 months, spanning from May 2019 to April 2020. For more detailed information on the bulls during the rearing phase, please check Polizel et al. [11].

The 63 young bulls were utilized for the finishing phase. These bulls were all provided with the same diet. Upon the conclusion of the finishing phase, the animals were slaughtered at the school slaughterhouse at FZEA, with an average body weight of 610 kg. More information about finishing phase can be found in Polizel et al. [12].

### 2.2. Feeding Management

The finishing phase extended for a duration of 112 days, with the initial 14 days allocated for the animals to their respective diets. Subsequently, for the remaining portion of this period, the animals were consistently provided with diets characterized by an inclusion of both protein and energy. The concentrated inclusion in each of the diets was set at 50%, 62%, and 73%, respectively. Throughout this phase, the animals were fed twice daily, with the first feeding at 7:00 a.m. constituting 60% of the total ration, and the second feeding at 11:00 a.m. making up the remaining 40% of the total ration. These feedings were administered in the form of total mixed ration.

The formulation of the diets adhered to the guidelines outlined in the NRC [13]. The proportions of ingredients used and the chemical composition of the diets are presented in Table 1.

### 2.3. Histological Assessments

#### 2.3.1. Ruminitis Incidence and Papillae Morphometric

Following the slaughter and evisceration of the animals, each thoroughly cleansed rumen was examined. The rumen epithelium was evaluated based on the presence of lesions (rumenitis) and structural irregularities (e.g., clumped papillae) in accordance with the criteria outlined by McManus et al. [14]. These assessments employed a grading scale ranging from 0 (indicating the absence of lesions or abnormalities) to 10 (representing severe ulcerative lesions). The morphometric variable under scrutiny was the average number of papillae. A proficient assessor conducted the measurement of the mean number of papillae in the entire rumen fragment.

Morphometric analysis adhered to the methodology delineated by Daniel et al. [15] and Resende et al. [16]. A specimen measuring 1 cm^2^ was collected from the cranial rumen sac and promptly submerged in a receptacle containing 70% alcohol. It was securely stored until the subsequent measurement phase. The morphometric variable subjected to analysis was the average number of papillae per 1 cm^2^. An adept evaluator performed the mean measurement of papillae across the entire rumen fragment.

#### 2.3.2. Papillae Microscopic Histological Measurement

Histological analysis was executed, with adaptations following the methodology described by Odongo et al. [17]. To facilitate this analysis, a 1-cm^2^ specimen was excised from the ventral sac of each rumen for subsequent histological evaluation. These histological sections were subjected to staining with hematoxylin and eosin, followed by embedding in paraffin wax and sectioning.

Morphometric measurements, including parameters such as papillae surface area and thickness of the keratinized layer (KLT), were determined. Specifically, four distinct papillae from each animal underwent these measurements. To achieve this, a computer-aided system for image analysis using digital microscopy was employed to examine the histology of samples (Leica DVM6, Leica Microsystems, Wetzlar, Germany).

#### 2.3.3. Cecum Morphometrics

The assessment of cecum lesions was conducted post-evisceration, whereby all thoroughly cleaned ceca were evaluated. The evaluation of cecum epithelium entailed a classification based on the presence of cecal wall inflammation, lesions, and petechiae. This classification was executed using a scale that ranged from 0 (denoting the absence of lesions) to 10 (indicating severe lesions), with adaptations drawn from the methodology originally described by Bigham and MCmanus [18]. Notably, all ceca were subject to scoring by a trained individual, and the final dataset was derived from the average of two independent scores.

Moreover, a 1-cm^2^ specimen was collected from the central region of the cecum epithelium for subsequent histological scrutiny. These specimens were thoughtfully preserved in a buffered 4% paraformaldehyde solution until further histological analyses, as per the approach outlined by Devant et al. [19]. The histological analysis of the cecum epithelium involved a series of steps, including tissue sample dehydration, embedding in paraffin wax, sectioning at 8 μm, and staining with hematoxylin and eosin.

Histological measurements, specifically focusing on parameters like crypt depth and goblet cells, were undertaken. To ensure a representative analysis, these measurements were conducted on 10% of the total number of crypts per animal, following the methodology by Pereira et al. [20]. This analysis was facilitated using a Leica Qwin Image Analyzer integrated within a Leica electron light microscope.

### 2.4. rRNA 16S Sequences Samples

The first PCR was performed for locus-specific amplification. Then, AMPure XP beads were used to purify the PCR reaction, and the size of the fragments generated in the PCR reaction was evaluated by agarose gel electrophoresis. The second PCR was performed to link the barcodes from the Nextera XT kit, and new PCR purification and library validation steps were performed. Subsequently, the libraries were quantified, so that all samples/libraries were equimolarly united into a single pool.

To introduce complexity into sequencing, a heterogeneous control, phi-X phage, was combined with the amplicon pool. Finally, the libraries and phi-X were denatured to allow sequencing.

The fecal samples of fifteen animals (five of each treatment) were collected during the feedlot confinement period for the finishing diet, all at the same instance. Rumen samples were obtained immediately after the animal’s slaughter from the same fifteen mentioned above. DNA extraction was carried out using the Macherey Nagel NucleoSpin Tissue^®^ commercial kit, and 16S sequencing was conducted using the V4 primers [21] on the MiSeq platform (Illumina, San Diego, CA, USA), following the manufacturer’s recommendations.

Library preparation followed Illumina recommendations. Locus-specific primers designed for amplifying archaea or bacteria targeted specific regions of the 16S rRNA, with overhang sequences of adapters included in the locus-specific primers.

### 2.5. Statistical Analysis

#### 2.5.1. Histological Analyses of Rumen and Cecum

The data underwent analysis using the statistical analysis system (SAS) [22]. Prior to conducting the analysis, assessment was made regarding the presence of discrepant data points, commonly referred to as outliers, and the normality of residual distributions. The Shapiro–Wilk test was employed to evaluate the normality assumptions. A homoscedasticity test was employed (Levene test) on residuals to evaluate the requisite assumptions of ANOVA. In cases where these assumptions were not met, appropriate data transformations were applied.

For the datasets pertaining to rumenitis, rumen morphometrics, and cecum morphometrics and histological measurements, mixed models (PROC MIXED) were employed. The sires were treated as a fixed effect, while animal and dam ages were incorporated as linear covariates. Significant differences between treatments were determined at a 5% level of significance using the Tukey–Kramer test.

In the case of scores, as they are qualitative variables, we performed a Chi-Square test (X^2^) in the Prisma^®^ software 1.0 to evaluate if the different scores’ frequencies were associated with the prenatal nutritional treatments.

#### 2.5.2. rRNA 16S Sequencing Analysis

In the domain of 16S sequencing analysis, all computational procedures were executed within the Rstudio software 2023.12.1+402 framework [23], leveraging the Bioconductor platform [24,25]. Raw sequencing data were processed using the DADA2 package [26]. This entailed quality control measures, sequence trimming, and the assignment of taxonomic classifications, all predicated on the SILVA database [27].

The ensuing dataset, comprising amplicon sequence bariants (ASVs), taxonomic information, animal-related metadata, sequence information, and phylogenetic annotations, was encapsulated within a phyloseq-class object, as facilitated by the phyloseq package [28]. After data integration, stringent outlier filtering was applied. This process involved the removal of phyla representing less than 0.01% of the total abundance, as well as the exclusion of taxa observed in less than 5% of the total samples.

The phyloseq-class object was subsequently transformed into a DGElist format through the utilization of the edgeR package [29]. This transformation served as a preparatory step for the calculation of differential taxonomic abundances across samples, employing the Limma Voom methodology [30]. Data visualization was undertaken using the ggplot2 package [31].

#### 2.5.3. Pearson’s Correlation Analysis

To identify the correlations between the histological assessments (characteristics of the rumen and cecum) and the 20 most abundant ASVs, we performed Pearson’s correlation analysis using the function “cor” in the R statistical environment. To visualize the results, we created heatmaps (ggcorrplot package) referring to the correlations found, highlighting the significant results (*p* < 0.05).

## 3. Results

### 3.1. Rumenitis and Cecum Cells Score

**Figure 1:**
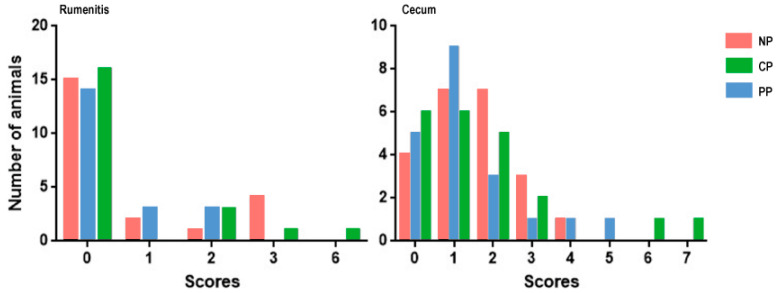
Rumenitis and cecum score of Nellore steers from different fetal programing.

Most animals included in this study received scores ranging from 0 to 2 for both rumenitis and cecum. While two animals from the CP treatment exhibited rumenitis and cecum scores exceeding 6.0, neither rumenitis nor cecum scores demonstrated a statistically significant effect, with *p*-values of 0.19 and 0.71, respectively. This is visually depicted in Figure 1 below.

The examination of morphometric characteristics in the rumen and cecum of Nellore cattle, in conjunction with a comparative assessment among different FP treatments, has yielded distinct outcomes, as succinctly summarized in Table 2.

In the cecum, animals subjected to the CP treatment exhibited a significant reduction in goblet cell count when contrasted with their NP counterparts. Moreover, the crypt depth in the cecum of CP-treated animals was notably diminished, bearing a *p*-value of 0.01.

Conversely, the utilization of the PP treatment was not statistically significant in crypt depth when compared to the NP treatment. Nevertheless, a reduction in goblet cell count was discerned in the cecum of PP-treated animals in comparison to those under the NP regimen.

Concerning the rumen, the PP treatment unveiled a notable escalation in rumen papillae quantity when juxtaposed with the NP treatment, aligning with a pattern akin to that observed in the CP treatment, a pattern substantiated by a *p*-value of 0.01. Nonetheless, no statistically significant distinctions were ascertained across the treatments regarding rumen papillae abundance or KLT (NP and PP).

### 3.2. Sequencing of Ruminal and Fecal Bacterial Communities

**Figure 2:**
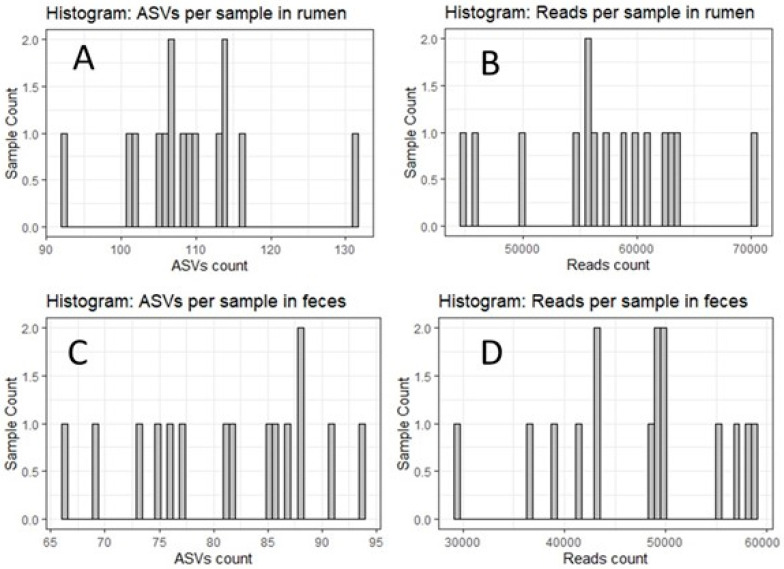
Histogram of sequencing reads and amplicon sequence variants (ASV) for rumen and fecal samples.

Utilizing the DADA2 software 1.25, which facilitated comprehensive quality control of 16S sequencing datasets, it became evident that samples derived from the rumen exhibited a notably higher richness and diversity of microbial populations when contrasted with their fecal counterparts. Within the ruminal ecosystem, an estimated range of 90 to 130 distinct amplicon sequence variants (ASVs) was discerned, as distributed across 45,000 to 70,000 sequencing reads, encompassing the entirety of the 15 sampled specimens. In striking contrast, the fecal samples contained a somewhat reduced spectrum of ASVs, numbering between 65 and 95, and were based on sequencing reads spanning 30,000 to 60,000 reads across the same set of 15 samples. This pattern is graphically illustrated in Figure 2 below.

**Figure 3:**
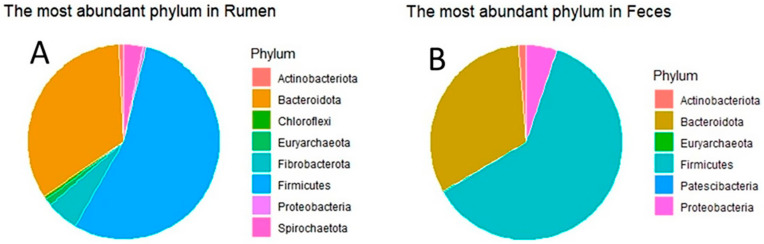
Pie chart representing abundance in sample collection sites: (**A**) rumen; (**B**) feces.

A scrutiny reveals the preeminent bacterial phyla populating the distinct ecological realms under investigation, as depicted in Figure 3. Each of these phyla makes a substantial contribution to the overall microbial abundance, with a total of eight and six dominant phyla discerned within the rumen and fecal ecosystems, respectively. Evidently, Firmicutes and *Bacteroidota* conspicuously surface as the predominant phyla in both habitats. Their eminent status is further accentuated by the shared presence of *Actinobacteriota*, *Euryarcheota*, and *Proteobacteria*.

Significantly, within the ruminal milieu, renowned for its heightened ASV diversity, as delineated in Figure 2, it is overtly manifest that these phyla also assume an augmented numerical preeminence. Most remarkably, the *Bacteroidota* phylum consistently represents approximately one-third of the aggregate abundances across both environmental settings.

The preeminent taxa, characterized by their abundance within the sampled specimens, are presented in the Table 3, specifically detailed in Supplementary Materials (SM S1 and S2). An investigation highlights that among these, the most represented genus and class inhabiting the gastrointestinal tract (GIT) of these animals are *Prevotella* and *Clostridia*, respectively. In the rumen, it is notably apparent that the taxonomic landscape encompasses additional taxa from diverse families, exemplifying a palpable manifestation of the heightened diversity inherent to this anatomical region. In stark contrast, the fecal environment distinctly exhibits a preponderance of the *Lachnospiraceae* family.

**Figure 4:**
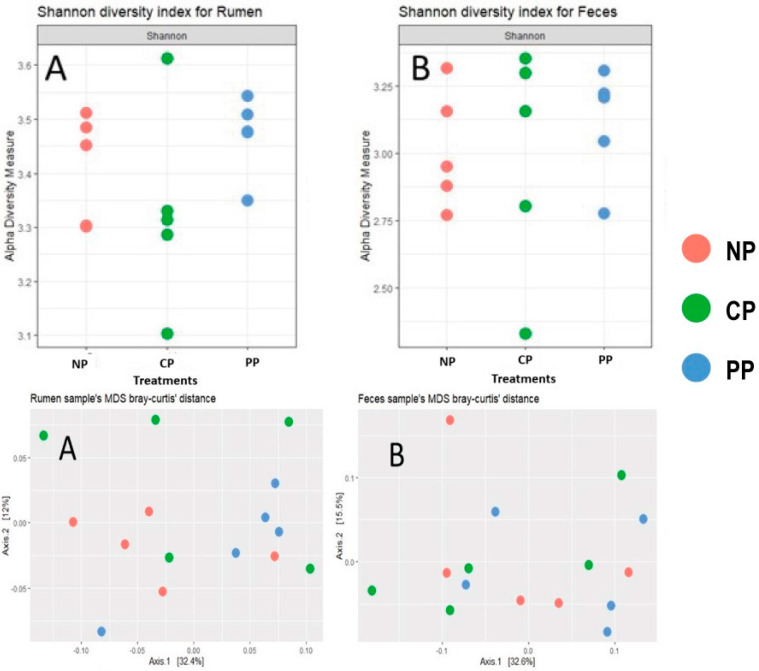
Shannon’s alpha diversity and Curtis beta diversity of rumen (**A**) and feces (**B**).

In the context of Shannon’s alpha diversity (Figure 4) across the three experimental treatments, an intriguing observation emerges. It is discerned that animals within the CP treatment exhibit an elevated diversity, reaching a value of 3.3, specifically in rumen samples when compared to fecal samples. However, it is important to underscore that this divergence did not manifest as a statistically significant difference among the treatments.

Beta diversity (Figure 4), encapsulating the distinctions between samples through the calculation of Bray-Curtis dissimilarity, disclosed intriguing patterns within both rumen and fecal domains. Although tentative clusters are discernible in the PP treatment, these groupings, when subjected to rigorous statistical scrutiny, did not attain statistical significance. Furthermore, the inter-treatment dissimilarities were remarkably inconspicuous. This phenomenon is vividly exemplified by the axes, where values of 32.4% and 12% are attributed to rumen samples, and 32.6% and 15.5% are attributed to fecal samples, attesting to the strikingly similar composition of microbial communities across the different FP treatments.

In this sense, Table 4 elucidates the temporal dynamics in the differential abundance of microorganisms within the rumen and fecal domains. Microorganisms were scrutinized for statistical significance, with particular attention given to those exhibiting a *p*-value threshold greater than or equal to 0.1. This evaluation unveiled noteworthy findings, with the rumen manifesting differential trends across five distinct microbial families, while the fecal environment showcased such trends within two unique families.

Prominently, the microorganisms that most conspicuously demonstrated a differential inclination among the various treatment groups belonged to the *Bacteroidales* BS11 family. This microbial family exhibited an adjusted *p*-value of 0.13, further accentuated by a *p*-value less than 0.01, thereby signifying its heightened abundance in untreated animals.

### 3.3. Pearson’s Correlation Analysis

**Figure 5:**
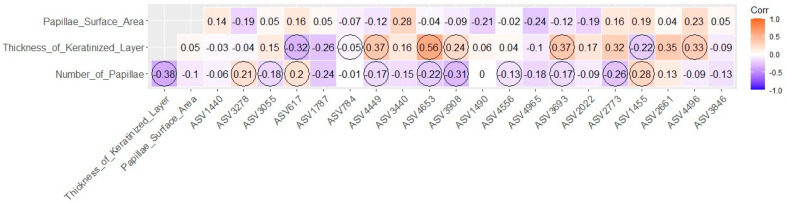
Correlations heatmap chart between the quantitative rumen histological traits and the top 20 most abundant ASVs. The significant correlations (*p* < 0.05) are circled.

According to the Figure 5, the heatmap showed several significant Pearson’s correlations between thickness of keratinized layer and the ASVs (n = 8); between the number of papillae and the ASVs (n = 11); and between the thickness of keratinized layer and number of papillae (r = −0.38).

**Figure 6:**
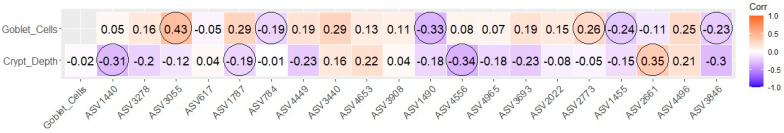
Correlations heatmap chart between the quantitative cecum histological traits and the top 20 most abundant ASVs. The significant correlations (*p* < 0.05) are circled.

Regarding Figure 6, the heatmap showed some significant Pearson’s correlations between goblet cells and the ASVs (n = 6); crypt depth and the ASVs (n = 4); and no significant correlation between the histological assessments.

## 4. Discussion

The current investigation stands as a pioneering endeavor, and the data it has unearthed hold the potential to catalyze novel research in the field. To date, a conspicuous gap exists in the realm of studies scrutinizing the epithelial characteristics of the rumen and cecum in the context of FP, although a recent study has been conducted involving goats, which delves into the exploration of microbial communities within the rumen and cecum from the fetal stage to adulthood [32]. The present study is distinct in its focus on bovine species, underscoring the unique dietary and physiological attributes that merit distinct scrutiny.

This underscores the pivotal role of maternal nutrition in molding the offspring’s productivity throughout its lifetime. Conversely, maternal nutritional constraints bear the potential to profoundly affect neonatal mortality rates, induce alterations in body composition, disrupt hormonal equilibrium, and even exert influence over organ development [33]. In a study involving sheep, it was observed that malnutrition adversely affected cecal microbial diversity, composition, and fermentation parameters. This, in turn, impeded intestinal immunological functions and hindered epithelial renewal [34].

Goblet cells are intricately linked to the secretion of mucus, serving as pivotal components in the gastrointestinal tract’s immunological defense mechanism. As elucidated by Birchenough et al. (2016) [35], goblet cells constitute the primary defense line of the intestinal mucosa, primarily owing to their secretion of mucin. An illustrative instance of the direct immune system regulation of goblet cells manifests in the hyperplasia and hypersecretion of mucus observed in response to parasitic helminth infections [36,37].

In the context of this study, Nellore cattle subjected to the NP treatment exhibited a higher goblet cell count, while those undergoing the PP and CP treatments displayed reduced goblet cell numbers. Furthermore, an increase in crypt depth was observed in the NP and PP treatments, a phenomenon often associated with heightened mucous secretion, as noted by Kotunia et al. (2006) [38]. This phenomenon could potentially explain the increased goblet cell count in the NP treatment but not in the PP treatment. Consequently, it is plausible to speculate that the findings of this study may be intricately linked to the maternal nutritional status during pregnancy, signifying the phenomenon of FP.

Fetal programming’s impact extends beyond skeletal muscle development in ruminant animals [6]. Originating from early human epidemiological research connecting low birth weight and maternal malnutrition to heightened adult disease risk, according to Barker et al. [39], the scope of FP in cattle encompasses not only muscle but also intestinal immunity [40]. Animals without good nutritional support during pregnancy may exhibit increased susceptibility to inflammation [41] and a heightened demand for mucus production.

The gastrointestinal tract development plays a pivotal role in the well-being of offspring from birth onwards, particularly concerning colostrum ingestion, given its enhanced stomach and intestinal epithelium, facilitating superior nutrient absorption [42]. A study on lambs subjected to FP revealed that those released from dietary restriction exhibited reduced immunoglobulin (IgG) intake, while lambs born to supplemented ewes displayed enhanced IgG absorption. This suggests that well-nourished gestational periods endow animals with a greater capacity to harness large molecules in the immediate postnatal phase [43]. A fully developed gastrointestinal tract equips offspring to maximize nutrient utilization [44].

Within the various compartments of the gastrointestinal tract, the present study focused on the evaluation of the rumen and cecum. In this context, the rumen exhibited significant differences in terms of papillae numbers, while the cecum demonstrated contrasting outcomes concerning crypt depth and caliciform cell abundance. Previous research encompassing an assessment of all gastrointestinal compartments in calves subjected to FP revealed a remarkable increase in small intestine length among animals whose cows’ received supplementation during pregnancy. This indicates that maternal supplementation influences the absorptive surface area in the intestine, facilitating greater nutrient uptake [45,46]. As exemplified in our current investigation, dams receiving supplementation during the final trimester of pregnancy foster enhanced development of their offspring’s cecal epithelium, thereby enabling heightened nutrient absorption in the intestine.

Dietary restriction can exert profound effects on the structural and functional development of visceral organs. As demonstrated in a study by Cavalcanti et al. (2014) [47], animals subjected to dietary restriction may experience significant alterations in the absorptive capacity of their gastrointestinal tract. In the context of the present investigation, it is crucial to emphasize that the animals involved were not subjected to dietary restriction. Consequently, no substantial variations in papillae thickness were observed. This outcome can plausibly be attributed to the application of nutritional restriction to the dams during the gestational period, which, in turn, may have had consequential repercussions on the developmental trajectory of their progeny.

According to a study conducting an assessment of the influence of maternal nutritional supplementation during the final trimester of gestation on the development of rumen papillae in sheep [48], there was an increase in the occurrence of ruminal papillae among lambs born to ewes that received supplementation during the latter stages of pregnancy. In concordance with these observations, the subjects enrolled in the present study and subjected to the PP treatment exhibited a statistically significant elevation in the number of rumen papillae in comparison to their counterparts undergoing alternative treatment protocols.

In the context of the present investigation, microscopic morphometric analysis of the rumen epithelium did not unveil significant differences between the treatment groups. However, a higher abundance of rumen papillae was distinctly evident within the PP treatment cohort when compared to the NP group, although no significant deviation was discerned in relation to the CP treatment. A wealth of scientific studies has underscored the profound influence of dietary factors on rumen epithelial characteristics. As elucidated by Dirksen, Liebich, and Mayer (1985) [49], the presence of an extensive population of rumen papillae serves to enhance the absorption of short-chain fatty acids (SCFA). Furthermore, it is well-documented that the degree of ruminal epithelial development is intrinsically linked to the speed of SCFA absorption, a phenomenon consistently corroborated by [50].

Nonetheless, it is imperative to acknowledge that this study was not expressly designed to evaluate SCFA production or absorption rates. Therefore, we must exercise caution when speculating about whether the observed increase in rumen papillae among PP-treated animals may translate to a heightened capacity for SCFA production or absorption. Moreover, the absence of statistically significant differences in microscopic measurements suggests that dietary composition may exert more influence on rumen epithelial development than the nutritional status of the dams during pregnancy.

The colonization of initial microorganisms in the rumen begins mainly during the prenatal phase, promoting a complex interaction between cows and their offspring in the uterine environment [51]. This interaction manifests itself as a remarkable similarity in the composition of the microbial communities found in both cows and calves, evident in meconium, the calf’s rumen fluid, and lasting until the weaning period [52]. After weaning, the stability of the microbial community critically depends on the dietary components provided to the animal throughout its life [53]. During the early stages of calf development, *fibrolytic* microorganisms of maternal origin are already discernible, which play the essential role of preparing the calf for effective digestion of fibrous material [54]. Thus, the cow contributes significantly to shaping the developmental trajectory of the calf’s gastrointestinal tract, thus highlighting the impact of FP in the early stages of an animal’s life.

The predominant members of the microbial community were with the phyla *Actinobacteriodota*, *Bacteriodota*, *Firmicutes*, *Chloroflexi*, *Proteobacteria*, *Prevotella*, and *Lachnospiraceae*. Notably, the abundance of Actinobacteria in rumen fluid suggests their potential role in the digestion of α-amylase and starch degradation, which may be attributed to their association with forage consumption in ruminant diets [55]. The dominant bacterial phyla within the rumen, such as *Bacteriodota*, *Firmicutes*, and *Chloroflexi*, have established connections with feed efficiency and weight gain in ruminants [56,57].

In the gastrointestinal tract, it is evident that the dietary components play a pivotal role in shaping the prevalence of bacterial phyla, alongside the specific gut compartments. The selective pressure of dietary factors is indeed a critical determinant for the relative abundance of microorganisms [58]. A pertinent example is the phylum *Proteobacteria*, which is more prevalent in grain-based diets [59].

Hence, the contrasting results observed in the rumen and feces underscore the significant impact of both diet composition and the localized microenvironment within the gastrointestinal tract, which influences the development of distinct microbial communities (Lopes et al. 2019 [60]). In healthy animals, *Prevotella* emerges as the dominant microbial genus in the gastrointestinal tract, which aligns with the findings in the present study. Additionally, the family *Lachnospiraceae* was the most abundant phyla in fecal samples, consistent with prior investigations involving Nellore cattle, highlighting their widespread presence across the gastrointestinal tract, this phylum possesses the ability to degrade fiber and proteins and is associated with enhanced feed efficiency [60].

Differential abundance analysis, performed through EdgeR’s limma voom methodology, is well-established in the context of both gene expression analysis [29,30] and its adaptation for metagenomic data [61]. This approach ensures the normalization of data within a specified statistical model, tailored for this study as a three-level treatment factor. Consequently, it effectively identifies the distinctive elements within each level of treatment. Notably, the *Bacteroidales* BS11 family exhibited a *p*-value below 0.01, with the lowest adjusted *p*-value indicative of a significant trend observed within the rumen samples. This microbial family has been linked to a diet characterized by a high content of hemicellulose and lignin and a low protein content in the Alaskan moose [62].

According to Schober et al. [63], correlations with values between ±0.40 and ±0.69 are considered moderate. In our study, we found only two significant moderate correlations; the others were weak or non-significant correlations at the 5% level of significance (*p* < 0.05). The strongest correlation observed in our results was between rumen keratin layer thickness and ASV4653, although it is still considered moderate (r = 0.56). The ASV4653, a member of the *Lachnospirales* order, has the potential to ferment butyrate and serve as a producer of rumen butyrate [64]. By redirecting hydrogen from reducing CO2 for methane formation to butyrate production, it may play a role in improving feed efficiency in cows [65]. Although prenatal nutritional treatments do not have an impact on the thickness of the keratin layer in the rumen, this characteristic is important as it presents the strongest correlation with an order of bacteria that performs essential functions related to rumen fermentation and feed efficiency. The second and last significant moderate correlation was identified between goblet cells and ASV3055 (r = 0.43). ASV3055 belongs to the order *Acidaminococcales* and the genus *Succiniclasticum*. Bacteria of this genus have functions of fermenting succinate to propionate to produce energy [66]. According to the results found, the NP group presented a greater quantity of boblet cells than the other groups, and the CP group presented the smallest quantity of goblet cells. As the correlation found was positive and moderate in relation to this variable and the genus of bacteria *Succiniclasticum*, we can state that the greater number of goblet cells can lead to a tendency towards a greater quantity of bacteria of the genus *Succiniclasticum*. Although we did not find differences in the abundance of bacteria of this genus, the results shown in this study corroborate the literature in terms of innovation and contribute to elucidating the molecular mechanisms of fetal programming in beef cattle.

## 5. Conclusions

This study offers a pioneering exploration of fetal programming’s implications for the gastrointestinal tract’s epithelial characteristics and microbial communities. Addressing a notable gap in research, it examines how maternal nutrition during pregnancy influences offspring organ development and intestinal immunity. Goblet cell counts and crypt depth variations in the gastrointestinal tract underscore the influence of FP. Ultimately, these findings contribute to our understanding of fetal programming’s broad-ranging implications for the gastrointestinal tract’s development, shedding light on both epithelial characteristics and microbial communities and their relevance in the context of maternal nutrition and offspring health.

## Figures and Tables

**Table 1:** Feed ingredients and chemical composition of the finishing diets fed to Nellore steers.

Diets	Initial	Intermediary	Finishing
Inclusion (%)	50	62	73
**Ingredients (% of DM)**			
Corn silage	50.00	38.75	27.50
Fine ground corn	34.90	50.88	67.05
Soybean meal	12.60	7.50	2.20
Urea	0.50	0.87	1.25
Flexbeef ^1^	0.66	-	1.00
Flexbeef MD ^2^	0.34	1.00	-
Flexbeef MAX ^3^	1.00	1.00	1.00
Monensin, mg	102.0	300.0	0.00
Virginiamycin, mg	250.0	250.0	250.0
**Chemical composition**			
DM ^4^ (%)	48.10	53.60	50.60
CP ^4^ (%DM)	15.00	14.00	13.00
RDP ^4^ (%DM)	10.31	10.18	10.00
NDF ^4^ (%DM)	36.50	31.10	25.80
NDFe ^4^ (%DM)	26.20	20.80	15.50
Ca ^4^ (%DM)	0.66	0.61	0.55
P ^4^ (%DM)	0.38	0.37	0.35
K ^4^ (%DM)	1.00	0.81	0.61
S ^4^ (%DM)	0.20	0.17	0.13
EE ^4^ (%DM)	3.18	3.43	3.68
TDN ^5^ (%DM)	71.00	73.6	76.2

^1^ Flexbeef, quantity per kg of product: 280 g of calcium (Ca); 15 g of phosphorus (P); 45 g of sulfur (S); 75 g of sodium (Na); 22.5 mg of cobalt (Co); 750 mg of cooper; 150 mg of fluorine (F); 25.5 mg of iodine (I); 155 g of magnesium (Mg); 1.005 mg of manganese (Mn); 7.5 mg of selenium (Se); 1.995 mg of zinc (Zn); and 142.5 UI of Vitamin A. ^2^ Flexbeef MD, quantity per kg of product: same composition of Flexbeef, differing only by the addition of 3000 mg of sodium monensin. ^3^ Flexbeef MAX: same composition of Flexbeef, differing only by the addition of 2500 mg of Virginiamycin. ^4^ Quantified through chemical analysis. ^5^ Value estimated by NRC (2016).

**Table 2:** Rumen and cecum morphometrics of Nellore steers from different fetal programing.

Variables	Treatments			Mean	SE	*p*-Value
	NP	PP	CP			
**Rumen measurements**						
*Macroscopic variables*						
Number of papillae, n	101.3 ^b^	122.9 ^a^	111.2 ^ab^	111.46	13.246	0.01
*Microscopic variables*						
Papillae thickness, µ	164.6	178.7	176.8	173.4	40.181	0.34
KLT, µ	12.55	12.75	12.19	12.49	2.144	0.63
**Cecum measurements**						
Crypt depth, µ	8.86 ^a^	10.40 ^a^	6.47 ^b^	8.57	2.637	<0.01
Goblet cells, n	33.77 ^a^	19.70 ^b^	11.91 ^c^	21.79	9.078	<0.01

SE: standard error; KLT: keratinized layer thickness; ^abc^: different letters on the same line differ significantly according to the Tuckey test at 5% of the significance level.

**Table 3:** Most abundant ASVs in 16S sequencing of rumen and feces.

ASV	Total	Kingdom	Phylum	Class	Order	Family	Genus
**Rumen**							
1440	119,904	*Bacteria*	*Bacteroidota*	*Bacteroidia*	*Bacteroidales*	*Prevotellaceae*	*Prevotella*
3278	69,851	*Bacteria*	*Firmicutes*	*Clostridia*	*Christensenellales*	*Christensenellaceae*	*R-7 Group*
3055	58,166	*Bacteria*	*Firmicutes*	*Negativicutes*	*Acidaminococcales*	*Acidaminococcaceae*	*Succiniclasticum*
617	49,365	*Bacteria*	*Bacteroidota*	*Bacteroidia*	*Bacteroidales*	*Rikenellaceae*	*RC9-gut Group*
1787	45,785	*Bacteria*	*Fibrobacterota*	*Fibrobacteria*	*Fibrobacterales*	*Fibrobacteraceae*	*Fibrobacter*
784	40,905	*Bacteria*	*Bacteroidota*	*Bacteroidia*	*Bacteroidales*	*F082*	*N/A*
4449	36,740	*Bacteria*	*Firmicutes*	*Clostridia*	*Lachnospirales*	*Lachnospiraceae*	*N/A*
3440	34,880	*Bacteria*	*Firmicutes*	*Clostridia*	*Oscillospirales*	*Oscillospiraceae*	*NK4A214 Group*
4653	34,174	*Bacteria*	*Firmicutes*	*Clostridia*	*Lachnospirales*	*Lachanospiraceae*	*NK3A20 Group*
3908	31,554	*Bacteria*	*Firmicutes*	*Clostridia*	*Oscillospirales*	*Ruminococcoceae*	*Runinococcus*
**Feces**							
1490	127,598	*Bacteria*	*Bacteroidota*	*Bacteroidia*	*Bacteroidales*	*Prevotellaceae*	*Prevotella*
4556	61,220	*Bacteria*	*Firmicutes*	*Clostridia*	*Lachnospirales*	*Lachnospiraceae*	*N/A*
4965	41,327	*Bacteria*	*Firmicutes*	*Clostridia*	*Lachnospirales*	*Lachnospiraceae*	*Blautia*
3693	39,565	*Bacteria*	*Firmicutes*	*Clostridia*	*Clostridiales*	*Clostridiaceae*	*Clostridium*
2022	39,074	*Bacteria*	*Firmicutes*	*Bacilli*	*Erysipelotrichales*	*Erysipelotrichaceae*	*Turicibacter*
2773	34,951	*Bacteria*	*Firmicutes*	*Clostridia*	*Tissierellales*	*Peptostreptococcaceae*	*Romboutsia*
1455	31,118	*Bacteria*	*Bacteroidota*	*Bacteroidia*	*Bacteroidales*	*Prevotellaceae*	*NA*
2661	30,781	*Bacteria*	*Proteobacteria*	*proteobacteria*	*Aeromonadales*	*Succinivibrionaceae*	*Succinivibrio*
4496	26,519	*Bacteria*	*Firmicutes*	*Clostridia*	*Lachnospirales*	*Lachnospiraceae*	*Agathobacter*
3846	25,090	*Bacteria*	*Firmicutes*	*Clostridia*	*Oscillospirales*	*Ruminococcaceae*	*Faecalibacterium*

ASVs (amplicon sequence variants): unique DNA sequences that represent an organism.

**Table 4:** Trend of differential abundance in rumen and feces. ASVs (amplicon sequence variants): unique DNA sequences that represent an organism. Adj. *p*-value: adjusted *p*-value.

ASV	*p*-Value	Adj. *p*-Value	Kingdom	Phylum	Class	Order	Family
**Rumen**							
695	0.0032615	0.1304584	*Bacteria*	*Bacteroidota*	*Bacteroidia*	*Bacteroidales*	*Bacteroidales_BS11*
3637	0.0285711	0.5406086	*Bacteria*	*Firmicutes*	*Clostridia*	*Clostridia_or*	*Hungateiclostridiaceae*
3908	0.0405456	0.5406086	*Bacteria*	*Firmicutes*	*Clostridia*	*Oscillospirales*	*Ruminococcaceae*
4260	0.0673547	0.6008402	*Bacteria*	*Firmicutes*	*Clostridia*	*Lachnospirales*	*Defluviitaleaceae*
3651	0.0751050	0.6008402	*Bacteria*	*Firmicutes*	*Clostridia*	*Monoglobales*	*Monoglobaceae*
**Feces**							
4556	0.0522220	0.9885435	*Bacteria*	*Firmicutes*	*Clostridia*	*Lactobacillales*	*Lachnospiraceae*
2058	0.0860555	0.9885435	*Bacteria*	*Firmicutes*	*Bacilli*	*Lactobacillales*	*Lachnospiraceae*

## Data Availability

The datasets generated during and/or analyzed during the current study are available from the corresponding author on reasonable request.

## Supplementary Materials

The following supporting information can be downloaded at: https://www.mdpi.com/article/10.3390/ani14060870/s1, Table S1: Feces microbiome relative abundance; Table S2: Rumen microbiome relative abundance.
